# Effects of spatial autocorrelation and sampling design on estimates of protected area effectiveness

**DOI:** 10.1111/cobi.13522

**Published:** 2020-08-13

**Authors:** Pablo Jose Negret, Moreno Di Marco, Laura J. Sonter, Jonathan Rhodes, Hugh P. Possingham, Martine Maron

**Affiliations:** ^1^ School of Earth and Environmental Sciences The University of Queensland Brisbane Qld 4072 Australia; ^2^ Centre for Biodiversity and Conservation Science The University of Queensland Brisbane Qld 4072 Australia; ^3^ Department of Biology and Biotechnologies Sapienza University of Rome Rome Italy; ^4^ The Nature Conservancy South Brisbane Queensland 4101 Australia

**Keywords:** Colombia, forest loss, general linear mixed models, human pressure, national park, simultaneous autoregressive models, statistical matching, Colombia, emparejamiento estadístico, modelos autorregresivos simultáneos, modelos mixtos lineales generalizados, parque natural, pérdida del bosque, presión humana, 噪音污染, 声污染, 干扰, 城市化, 野生生物

## Abstract

Estimating the effectiveness of protected areas (PAs) in reducing deforestation is useful to support decisions on whether to invest in better management of areas already protected or to create new ones. Statistical matching is commonly used to assess this effectiveness, but spatial autocorrelation and regional differences in protection effectiveness are frequently overlooked. Using Colombia as a case study, we employed statistical matching to account for confounding factors in park location and accounted for for spatial autocorrelation to determine statistical significance. We compared the performance of different matching procedures—ways of generating matching pairs at different scales—in estimating PA effectiveness. Differences in matching procedures affected covariate similarity between matched pairs (balance) and estimates of PA effectiveness in reducing deforestation. Independent matching yielded the greatest balance. On average 95% of variables in each region were balanced with independent matching, whereas 33% of variables were balanced when using the method that performed worst. The best estimates suggested that average deforestation inside protected areas in Colombia was 40% lower than in matched sites. Protection significantly reduced deforestation, but PA effectiveness differed among regions. Protected areas in Caribe were the most effective, whereas those in Orinoco and Pacific were least effective. Our results demonstrate that accounting for spatial autocorrelation and using independent matching for each subset of data is needed to infer the effectiveness of protection in reducing deforestation. Not accounting for spatial autocorrelation can distort the assessment of protection effectiveness, increasing type I and II errors and inflating effect size. Our method allowed improved estimates of protection effectiveness across scales and under different conditions and can be applied to other regions to effectively assess PA performance.

## Abstract 2

*Article impact statement*: Spatial autocorrelation and sampling design can distort estimations of protected area effectiveness at reducing forest loss.

## Abstract (es)

Efectos de la Autocorrelación Espacial y el Diseño del Muestreo sobre las Estimaciones de la Efectividad de Áreas Protegidas

**Resumen**

La estimación de la efectividad de las áreas protegidas (AP) para reducir la deforestación es útil al momento de respaldar las decisiones que eligen entre invertir en un mejor manejo de las áreas ya protegidas o crear áreas nuevas. El emparejamiento estadístico es la herramienta utilizada con mayor frecuencia para evaluar esta efectividad, pero casi siempre se ignora la autocorrelación especial y las diferencias regionales en la efectividad de la protección. Con Colombia como caso de estudio, empleamos un emparejamiento estadístico para controlar el efecto de factores relacionados con la ubicación la ubicación de los parques y he incluimos el efecto de la autocorrelación especial para determinar la significancia estadística. Comparamos el desempeño de los diferentes procedimientos de emparejamiento – las maneras de generar pares a diferentes escalas – en la estimación de la efectividad de las AP. Las diferencias en los procedimientos de emparejamiento afectaron la similitud de la covarianza entre los pares emparejados (balance) y la estimación de la efectividad de las AP en la reducción de la deforestación. El emparejamiento independiente produjo el mayor balance. En promedio, el 95% de las variables en cada región estuvo balanceado con el emparejamiento independiente, mientras que el 24% de las variables estuvo balanceado cuando se usó el método con el peor desempeño. Las mejores estimaciones sugieren que la deforestación media dentro de las áreas protegidas en Colombia era 40% menor que en los sitios emparejados emparejados. La protección redujo significativamente la deforestación, aunque la efectividad de las AP difirió entre las regiones. Las AP en la región Caribe fueron las más efectivas, mientras que aquellas en la Orinoquía y el Pacífico fueron las menos efectivas. Nuestros resultados demuestran que se necesita considerar la autocorrelación espacial y usar el emparejamiento independiente para cada subconjunto de datos para inferir la efectividad de la protección en la reducción de la deforestación. Si no se considera la autocorrelación espacial, se pueden distorsionar los estimativos de la efectividad de la protección, incrementando los errores de tipo I y II e inflando el tamaño del efecto. Nuestro método permitió obtener mejores estimaciones de la efectividad de la protección en todas las escalas y bajo diferentes condiciones y puede aplicarse a otras regiones para evaluar de manera efectiva el desempeño de las AP.

## 【摘要】

人为噪音是一种全球广泛存在的感官污染, 它被认为对野生动物的功能、种群和生理都有潜在的负面影响。人口增长以及城市化、交通和资源开采等方面的相关变化都会制造人为噪音, 且预计在未来几十年还会增加。相应地, 野生动物接触人为噪音的机会也预计会增加。通过实地研究收集的数据对于进一步了解人类活动对野生动物的实际影响具有独特的重要意义。因此, 我们对 2008 年至 2018 年发表的关于人为噪音影响的实地调查的文献进行了系统综述。我们评估了发表指标 (如发表率和期刊类型) 、研究的地理分布、研究主题和使用的方法。在我们评估涵盖的时间段内, 研究活动有显著增加。然而, 研究存在明显的地理偏向, 大多数在北美或欧洲进行, 且明显聚焦于陆地环境。陆地环境的研究中只有不到五分之一位于可能在 2030 年前发生城市化的农村地区, 这意味着最有可能受到未来变化影响的生态系统的数据没有得到收集。我们发现, 调查研究的类群也存在偏向性, 大多数研究是在鸟类和水生哺乳动物中进行的, 而陆生哺乳动物、爬行动物、两栖动物、鱼类和无脊椎动物得到的关注有限。尽管有证据表明动物主要的活动模式是夜间活动, 然而几乎所有陆地环境的研究只调查了昼间活动物种。近一半的研究调查了道路或城市噪音的影响; 大量研究仅限于功能性影响, 而非生理或种群方面的影响。很少有研究通过实验探讨长期暴露于人为噪音中的影响或是长期暴露后产生的效应, 也很少有研究对多种噪音类型或水平进行比较。弥补这些知识空缺对于成功管理不断加剧的野生动物暴露于人为噪音的影响至关重要。**【翻译: 胡怡思; 审校: 聂永刚】**

## Introduction

Measuring the effectiveness of protected areas (PAs) is important to conservation (Andam et al. 2008; Maron et al. 2013; Watson et al. 2014). Protected areas are fundamental to maintaining habitats and species diversity (Geldmann et al. 2013; Heino et al. 2015; Barnes et al. 2016). However, the extent to which PAs deliver conservation outcomes is debated, particularly in relation to preventing loss of natural ecosystems (Geldmann et al. 2013, 2019; Heino et al. 2015; Jones et al. 2018). One‐third of the extent of PAs worldwide are under intense human pressure (Jones et al. 2018), three‐quarters of the global land area currently protected lack resources to function properly (Coad et al. 2019), and PA downgrading, downsizing, and degazettement are widespread (Mascia & Pailler 2011). However, if pressures are reduced by protecting an area, then even a PA with declining biodiversity might be judged effective. Conversely, a PA that would not have lost biodiversity even if it were unprotected cannot be considered effective (Joppa et al. 2008; Ferraro, 2009).

Frequently, the effectiveness of PAs at preventing decline of forested ecosystems has been measured by comparing forest loss inside PAs with forest loss in the area surrounding the PA (Joppa et al. 2008; Geldmann et al. 2013; Heino et al. 2015). However, these methods are usually biased toward overestimating the effectiveness of protection because PAs are not randomly distributed across landscapes (Andam et al., 2008; Joppa & Pfaff, 2011). For example, PAs are often located far from roads and cities, where slope and elevation are high and agricultural productivity is low (Pressey et al. 2002; Joppa & Pfaff, 2009; Forero‐Medina & Joppa 2010), and deforestation is less likely to occur regardless of protection status (Joppa & Pfaff, 2009). Therefore, comparing such locations with surrounding areas likely shows a difference in deforestation rates, regardless of whether or not the area is protected.

Statistical matching improves understanding of the true effectiveness of PAs because it can be used to compare treatment (i.e., protected) sites with matched control sites (i.e., unprotected areas with characteristics similar to treatment sites) (Andam et al. 2008; Joppa & Pfaff 2011; Morgan & Winship 2015). This method has been used to estimate the effectiveness of PAs in preventing forest loss while accounting for nonrandom spatial allocation of PAs (Andam et al., 2008; Ferraro, 2009; Joppa & Pfaff, 2011; Bowker et al. 2017). However, the method's application varies, especially relative to how effectiveness within regional subsamples is estimated. For example, some researchers examine subsets of matched pairs from a larger‐scale matching analysis to make additional inferences for subregions (Blackman et al. 2015; Geldmann et al. 2019), whereas others use independent matching for each subregion analyzed (Zhao et al. 2019).

The possible presence of spatial autocorrelation in forest loss rates and its effect on estimates of protected area effectiveness is often overlooked when using statistical matching (e.g., Andam et al. 2008; Joppa & Pfaff 2011; Geldmann et al. 2019). A way to account for spatial autocorrelation in these analyses is by spacing sampling units at a certain distance (Gaveau et al. 2013; Bowker et al. 2017; Zhao et al. 2019), but the effectiveness of this procedure is rarely reported. Spatial autocorrelation is a well‐known problem that violates the assumption of data independence, and its presence inflates type I and II error rates (Mets et al. 2017; Crabot et al. 2019).

We assessed the performance of different matching procedures—ways of generating matching pairs at different scales—and the influence of spatial autocorrelation on estimates of PA effectiveness with Colombia as a case study. Colombia has >60 million ha of forest that extend over 58% of its area (World Resources Institute 2018) and is one of the most biodiverse countries on Earth (supporting an estimated 10% of global biodiversity) (Romero et al. 2008). The country faces major land‐transformation pressures (Etter & van Wyngaarden 2000), and deforestation has increased following the recent peace accord with FARC (Negret et al. 2017; Clerici et al. 2018). This makes PA effectiveness an essential element for the future of the country's biodiversity.

We evaluated the performance of PAs at national and regional levels and used different approaches to account for confounding factors in park location and spatial autocorrelation. We sought to inform decisions on whether to invest in better management of already‐protected areas or in creation of new ones (Adams et al. 2019) and decisions on where improved management is needed most (Coad et al. 2019).

## Methods

### Study Area

Colombia has a continental area of approximately 1,142,000 km². Climate is tropical, although temperatures vary widely with elevation. To avoid biodiversity loss the government has relied primarily on creation of PAs. To date, the country's PA network covers >13.5% of Colombia's continental area, and expansion is planned (Colombia Natural National Parks 2019). However, there is little information on the performance of Colombian PAs in preventing forest loss. Heino et al. (2015) showed that considerable forest loss occurs inside PAs and that observed rates of loss are lower for PAs in the Amazon than for PAs in the Andes. They did not compare these rates of deforestation with deforestation in similar unprotected areas, so the effectiveness of the PAs remains unknown.

**Figure 1:**
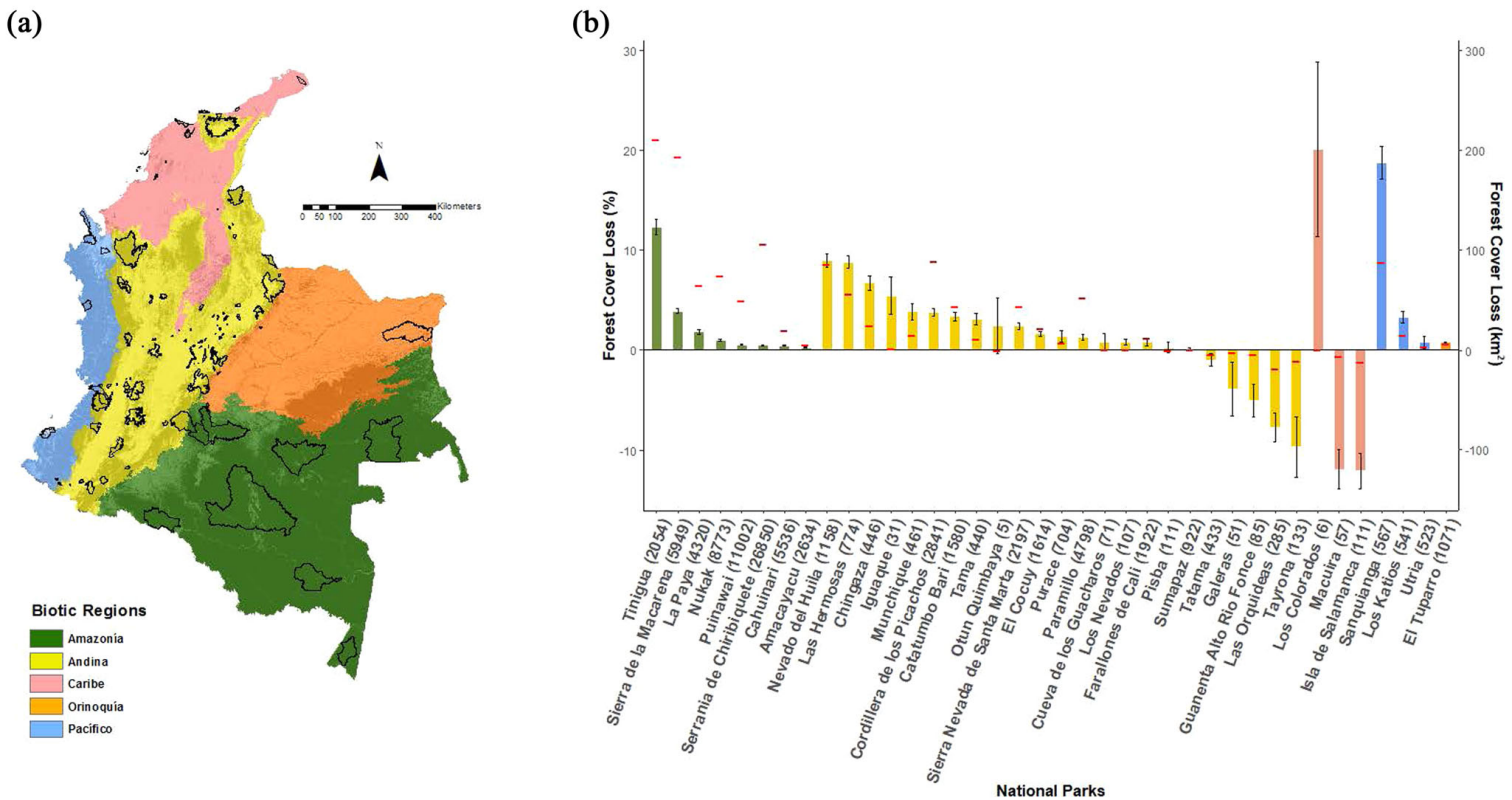
(a) Biotic regions of Colombia and the protected areas (PAs) (Amazon, 8 PAs; Andes, 85; Caribe, 8; Orinoquia, 6; Pacific, 9; dark shading, forest cover in 2000; pale shading, other land cover) and (b) average percent forest loss from 2000 to 2015 in each Colombian national park (*n* = 38) (negative values, forest gain; red horizontal lines, area lost from 2000 to 2015; black vertical lines, 95% CI; numbers in parentheses, square kilometers of forest cover inside each national park in 2000).

To assess the effectiveness of PAs in preventing net forest loss, we considered PAs in Colombia established before 2000 (Fig. 1), a total of 116 PAs covering 11,040 km². This area represents 9.8% of the country's continental area and 15% of its forest cover. Spatial boundaries, date of establishment, and management categories were obtained from the Colombian National System of Protected Areas (Colombia Natural National Parks 2019) and the World Database on Protected Areas (Protected Planet 2018).

### Forest Cover and Drivers of Forest Loss

To measure forest loss within and outside of PAs, we used forest‐cover maps for 2000 and 2015 from the Colombian Institute of Hydrology, Meteorology and Environmental Studies IDEAM (Galindo et al. 2014). The IDEAM maps have a 1‐ha resolution and define forest as land with a minimum tree canopy density of 30% and a minimum canopy height of 5 m. Tree cover from commercial forest plantations, palm crops, and trees planted for agricultural production was excluded (Galindo et al. 2014).

Due to clouds, shadows, and other data errors, IDEAM data were missing information on cells for approximately 5.8% of Colombia's area for the analyzed years, so we used posterior processing to reduce the proportion of cells with no information. We assessed the classification for 1 year on either side of each missing data cell. If a missing data cell was classified as forest in both the previous and following years, then the cell was classified as forest in the analyzed year. Similarly, if a missing data cell was classified as nonforest in both the previous and following years, it was classified as nonforest in the analyzed year. This process reduced the total area with no information to 1.1% of Colombia. Then we generated a 1‐km² grid covering the country and calculated the average proportion of forest cover for each cell for 2000 and 2015. The proportion of forest‐cover loss for each cell was calculated as the difference between proportion of forest cover in 2000 and 2015. Net proportion of forest loss was calculated for each PA, the whole PA network, each biotic region, and each International Union for Conservation of Nature (IUCN) protection category (IUCN 2017).

We obtained data on spatial variables associated with deforestation and park location in Colombia (Forero‐Medina & Joppa 2010; Negret et al. 2019) including initial forest cover; biotic regions; departments; population density; intensity of armed conflict; distance to major rivers, mines, oil wells, coca plantations, paved road, and unpaved road; and elevation. We used slope as a surrogate for land‐use potential because, for example, high agricultural potential is associated with low slope (Pressey et al. 2002; Forero‐Medina & Joppa 2010) (Supporting Information). Biotic regions and departments were also used as covariates and were transformed to dummy variables for posterior analysis. We included all covariates in the matching analysis to maximize similarity between matched pairs (Stuart & Rubin 2011; Schleicher et al. 2019). All variables were resampled to 1‐km² resolution. This resolution was a compromise between maintaining a low resolution for the forest data and the coarse resolution of other variables. Albeit not high in absolute terms, this resolution is relatively high compared with other studies (Armenteras et al. 2015; Brun et al. 2015).

### Statistical Matching

To estimate the effectiveness of the PA system in reducing net forest loss at the national scale, we used propensity‐score matching to create control groups for each PA cell in the country (Andam et al., 2008; Joppa & Pfaff, 2011). Propensity‐score matching is one of the most commonly used matching approaches in conservation (Schleicher et al. 2019), and it outperforms other commonly used methods, such as the Mahalanobis metric, when there are large numbers of covariates (Stuart & Rubin 2011). For each 1‐km² grid cell, we extracted values of variables associated with deforestation and park location. This was done both for the treatment group (all cells within PAs established before 2000) and the potential control group (cells in areas that had never been protected). We used the MatchIt package (Ho et al. 2011) in R (version 3.5.1) to pair treatment and control cells with similar values for each of the covariates. Matching was done without exact matching for any variable. We calculated covariate means for treatment and control observations before matching and used the nearest neighbor method to select matched controls that minimized differences (Andam et al. 2008; Joppa & Pfaff 2011; Sonter et al. 2019). We matched each treatment observation to a unique control observation to ensure each PA cell was paired with only 1 control cell that had not been matched previously. Matched pairs of cells had to be within 0.25 SD of the propensity scores (Blackman et al. 2015).

### Accounting for spatial autocorrelation

To assess the effect of spatial autocorrelation on the estimated effect of protection, we tested a generalized linear mixed model (GLMM) with forest‐cover loss as the response variable, protection as the predictor variable, and municipality and pair identification (matching pairs resulting from the matching process) as random effects (model 1) (Tognelli & Kelt 2004; Ver Hoef et al. 2018); a modified version of model 1 with a term to account for spatial autocorrelation that reflected the average net forest loss within 30 km around each record (model 2); a modified version of model 1 with 2 terms to account for spatial autocorrelation that reflected the average net forest loss within 30 and 5 km around each record (model 3); and a simultaneous autoregressive model (SAR) (Ver Hoef et al. 2018) with the same structure as model 1 plus a neighborhood contiguity weighted matrix (weighted values for each record up to 30 km) as the autoregressive component (Ver Hoef et al. 2018) (model 4). We compared the extent to which each method reduced residual spatial autocorrelation and the differences in their estimated effect of protection. To estimate the models’ respective capacities to account for residual spatial autocorrelation, we produced a Moran's *I* plot for 10,000 randomly selected residual values from both treatment and control cells from each model (Tognelli & Kelt 2004).

To reduce the computational complexity of the SAR (model 4) when applying the neighborhood contiguity weighted matrix, we randomly selected a subsample of 10,000 cells for modeling and plotted Moran's *I* for the residuals. The size of the buffer for model 2 and the distance for the weighted matrix in model 4 was 5 km less than the distance at which spatial autocorrelation was not different from 0 in model 1. The size of the second buffer for model 3 was 5 km less than the distance at which spatial autocorrelation was not different from 0 in model 2. The GLMM models were built using the package lmer4 (version 1.1 [Bolker 2019]). The SAR was implemented as a spatial autoregressive lagerror model, built using the function errorsarlm from the package spdep’(version 1.1 [Bivand 2019]), with a spatial link matrix generated using a weighting method that assigns higher leverage to spatial objects with few connections (W scheme) (Tiefelsdorf et al. 1999). All analyses were implemented in R statistical language version 3.5.1 (R Development Core Team 2018).

### Regional Analyses

The drivers of deforestation and spatial allocation of PAs likely vary among regions and so might the effectiveness of PAs. Therefore, we also evaluated PA effectiveness at a regional scale. For this, we used three different matching approaches used in other studies and compared their performance in generating matched pairs that were similar in terms of the covariates.

The first approach was a matching analysis at a national scale, as described above, for which we compared the subsets of protected cells in each region with their respective matched controls (subsetting) (Blackman et al. 2015). The second approach was a matching analysis at a national scale with exact matching for regions to ensure each matched pair belonged to the same biotic region followed by a separate assessment of each region's protected cells and their respective matched controls (exact subsetting) (Nelson & Chomitz 2011; Bowker et al. 2017; Geldmann et al. 2019). The third approach was an independent matching procedure for each region (submatching) (Zhao et al. 2019).To compare the performance of these three procedures, we calculated indices of covariate imbalance for each variable before and after matching through an estimation of normalized difference (Stuart & Rubin 2011; Olmos & Govindasamy 2015). Absolute scores >25% are considered an indication of a possible imbalance for that specific variable (Stuart & Rubin 2011; Olmos & Govindasamy 2015).

### Estimating Effectiveness of PAs

We calculated the average forest loss for each individual protected area and then aggregated it for the whole country, across different biotic regions, and across different types of PAs (as classified by IUCN). We used the matched pairs resulting from the most effective matching procedure to model the effectiveness of protection in each biotic region. We used the best model accounting for spatial autocorrelation to calculate the effectiveness of protection at national and regional levels.

## Results

### Loss of Forest Cover Inside PAs

**Figure 2:**
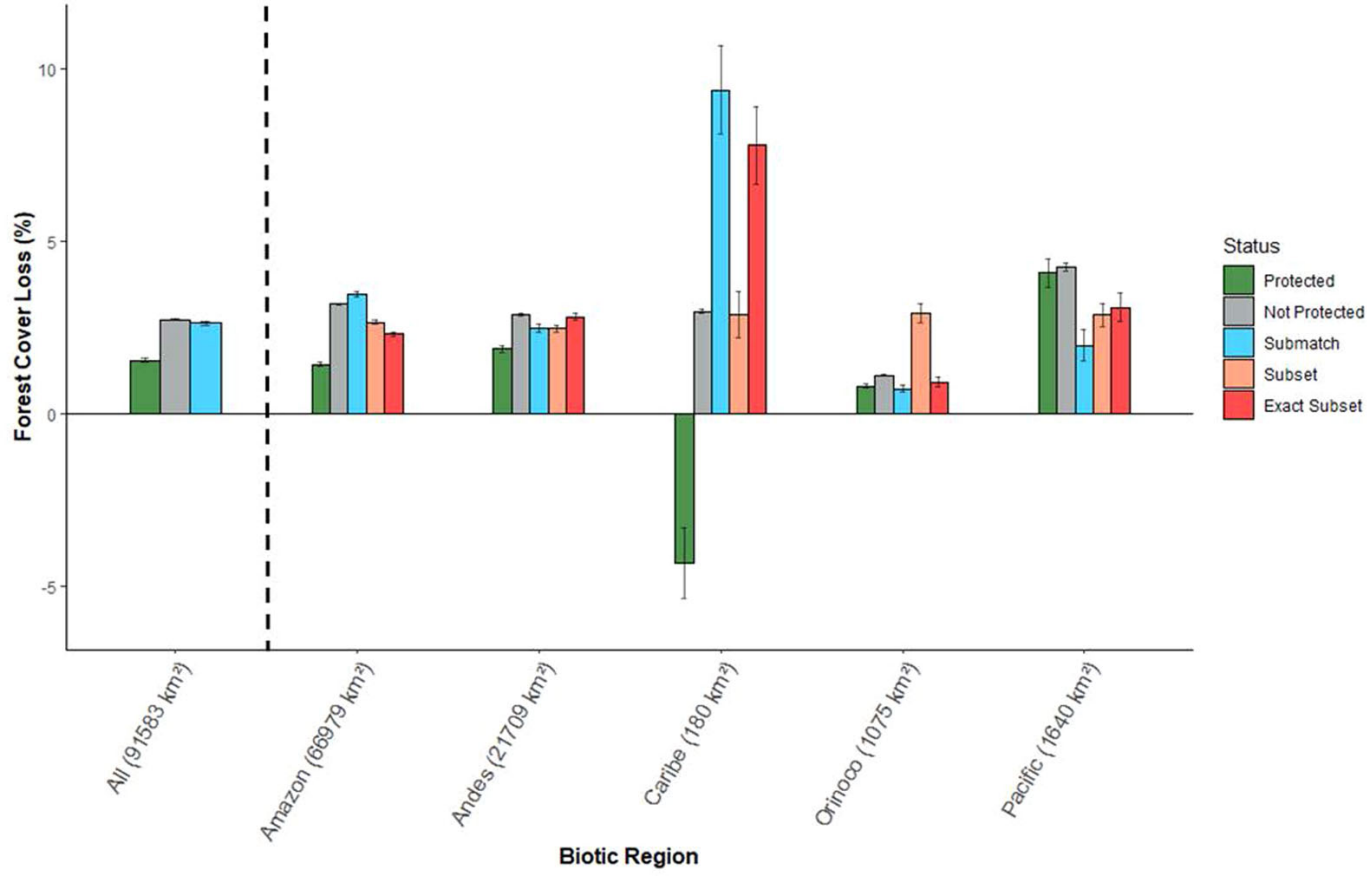
Average percent forest loss in protected areas (PAs) relative to overall forest loss outside PAs in Colombia and in matched unprotected control sites (numbers in parentheses, square kilometers of PA with forest cover for each biotic region in 2000; negative values, forest gain; green bars, loss inside PAs; gray bars, loss in unprotected areas; red, pink, and blue bars, loss in matched control sites based on different matching procedures; black lines, 95% CI).

Net forest loss from 2000 to 2015 in the PA network was 1.54% (Fig. 2) and was distributed across 82% of national parks (Fig. 1). Tinigua, Nevado del Huila, Las Hermosas, Los Colorados, and Sanquianga were the PAs with the highest proportional net forest loss (>10% each) (Fig. 1b). Overall deforestation outside PAs over the same period was 2.72% (Fig. 2).

Forest was lost in PAs in all regions, with the exception of Caribe, where PAs gained forest (average 4.3% gain) (Fig. 2). Protected areas in the Pacific region had the highest proportion of forest loss (average 4.1% loss) for that period (Fig. 2). Prematching comparisons showed areas that were not protected had higher average loss than protected area overall and within each region (Fig. 2). When PAs were aggregated by IUCN protection category, those in categories I (*n* = 2), II (*n* = 31), and IV (*n* = 7) on average lost forest cover, whereas those in categories III (*n* = 1) and VI (*n* = 75) gained forest cover (Supporting Information).

### Spatial Autocorrelation

**Figure 3:**
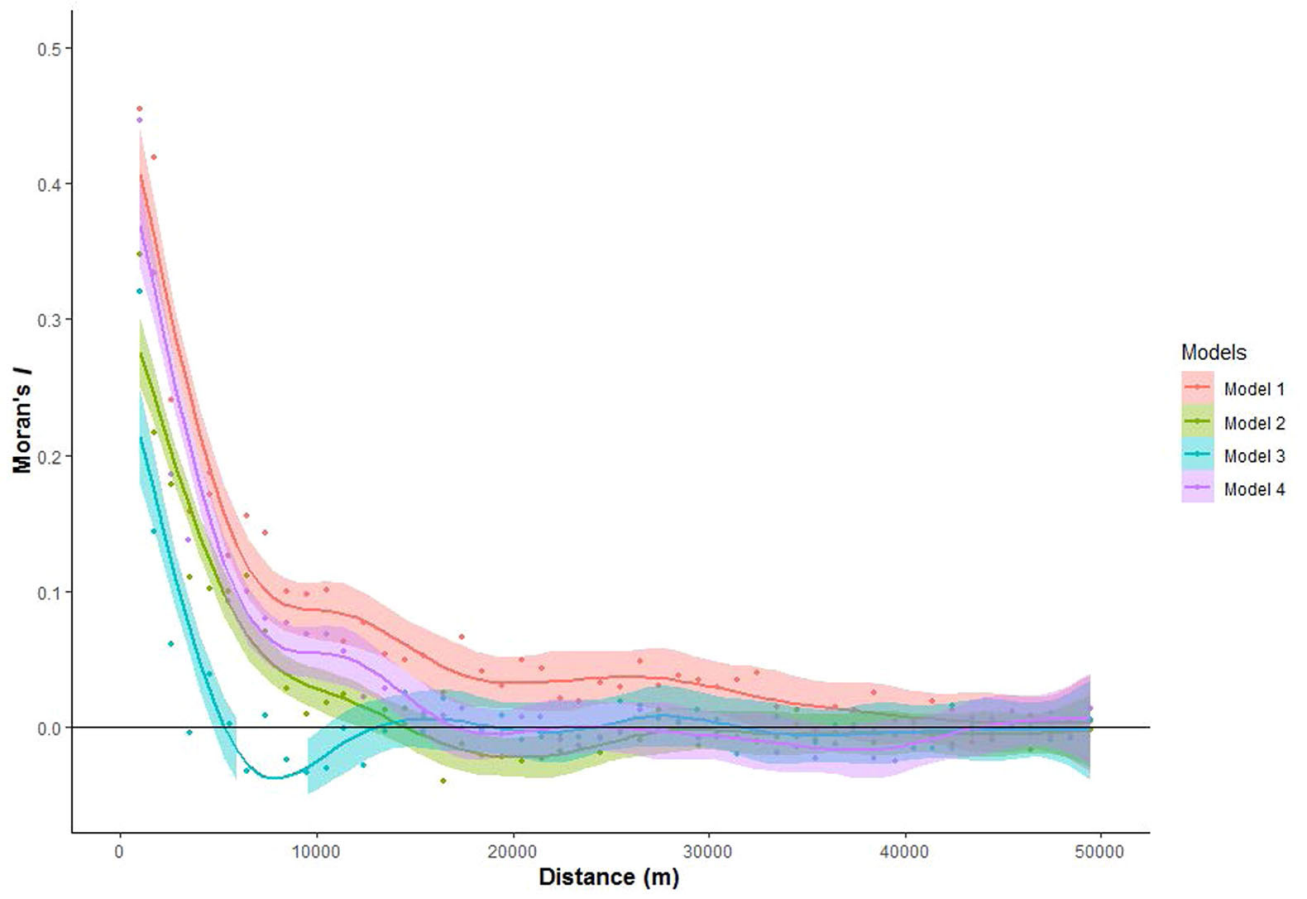
Moran's index (shading, 95% CI) for 10,000 randomly selected residual values from 4 different models of the effect of protection preventing deforestation (model 1, general linear mix model, forest‐cover change as response variable, protection as predictor variable, municipality as random effect; model 2, as for model 1 but with average deforestation in a buffer of 30 km around each record; model 3, as for model 1 but with average deforestation in a buffer of 30 and 5 km around each record; model 4, spatial autoregressive model with the same structure as model 1 and a neighborhood contiguity weighted matrix with weighted values for each record up to a distance of 30 km as the autoregressive component).

The Moran's *I* plot for the residuals of model 1 (the GLMM model) indicated significant spatial autocorrelation up to approximately 35 km (Fig. 3). Residuals of model 2 (GLMM with forest loss in buffer) showed significant spatial autocorrelation up to 10 km (Fig. 3). Residuals of model 3 (GLMM with two buffers of forest loss) showed significant spatial autocorrelation up to 5 km; mean values were <0.2 (Fig. 3). Model 4 (SAR) had significant residual spatial autocorrelation up to 15 km (Fig. 3). Model 3 accounted for spatial autocorrelation the best. None entirely eliminated spatial autocorrelation (Fig. 3).

Model 4 was excluded from the comparison of the coefficients and statistical significance of the effect of protection on forest loss because it required an exceptional computational complexity and its performance was the second poorest. The magnitude of the effect of protection decreased with the effectiveness of the model in accounting for spatial autocorrelation (Table 1). At a national scale, the negative effect of protection on forest loss was statistically significant for the three different models (Table 1). At a regional level, protection had a negative effect on forest loss for the Andes, Caribe, and Amazon, and this effect was statistically significant for all models except model 1 in the Andes region (Table 1). For the Pacific and Orinoco regions, protection did not significantly affect forest loss with model 1, but with model 3 this effect was positive and significant (Table 1).

**Table 1:** Coefficients and statistical significance of effectiveness of protected areas (PA) in reducing deforestation for the entire PA network and the network within each biotic region in Colombia relative to matched unprotected sites from the 3 modeling procedures.^a^

	Model 1	Model 2	Model 3
All area protected	−1.09^**^	−0.54^**^	−0.07^b^*
Biotic Region			
Andes	−0.5	−0.39**	−0.06 b**
Caribe	−11.58**	−10.55**	−1.82^b^*
Amazon	−1.90**	−0.51**	−0.08*
Pacific	−0.21	0.19	0.54^b^*
Orinoco	−0.03	0.34**	0.35 b**

a The coefficients of protected area effectiveness for each biotic region were from submatching, which was the matching procedure that generated the most adequate matchings at the regional level. Significance: *p < 0.05, **p < 0.01. Models are described fully in Methods.

b Coefficients from the model that better accounted for spatial autocorrelation.

### Statistical Matching

A comparison of cells in‐ and outside the PA network showed a propensity‐score imbalance (absolute scores >25%) for several variables, including initial forest cover, elevation, distance to nearest paved road, distance to mining, and distance to nearest oil well. After the initial national‐level matching analysis, all imbalances were <25%, an acceptable level (Stuart & Rubin 2011; Olmos & Govindasamy 2015) (Table 2 & Supporting Information).

**Table 2:** Indices of covariate imbalance for the principal variables associated with deforestation and park location in Colombia before and after the matching process for an initial matching analysis at a national scale and different matching procedures at the regional scale

		All	Amazon			Andes			Caribe			Orinoquia			Pacific		
			after matching			after matching			after matching			after matching			after matching		
	Before Matching	after matching	subsetting	exact subsetting	sub matching	subsetting	exact subsetting	sub matching	subsetting	exact subsetting	sub matching	subsetting	exact subsetting	sub matching	subsetting	exact subsetting	sub matching
Initial forest cover	75.7 a	3.7	36.6 a	5.6	3.7	50.0 a	12.7	1.6	29.9 a	30.6 a	16.2	61.7 a	5.4	3.2	53.3 a	15.2	3.2
Elevation	26.4 a	13.4	68.6 a	29.8 a	13.4	124.6 a	4.4	3.1	83.5 a	1.0	2.9	66.7 a	17.0	0.5	57.1 a	0.5	5.4
Slope	17.0	8.4	55.9 a	16.5	8.4	112.5 a	2.9	5.9	26.4 a	9.2	6.8	68.7 a	9.9	0.1	24.1	2.1	5.5
Population density	2.9	1.3	17.1	6.8	1.3	21.2	7.3	3.5	4.6	22.3	9.1	27.4 a	36.5 a	2.4	11.1	6.2	4.7
Rivers	16.4	3.7	3.3	18.0	3.7	12.5	60.5 a	0.2	14.2	46.3 a	12.8	54.4 a	143.8 a	5.0	81.3 a	3.0	1.5
Paved roads	25.7 a	5.1	27.6 a	1.0	5.1	115.3 a	41.5 a	29.7 a	68.0 a	85.7 a	12.1	225.2 a	65.4 a	0.7	78.9 a	3.1	2.2
Unpaved roads	23.9	0.8	38.3 a	3.7	0.8	94.6 a	0.7	29.4 a	51.1 a	22.9	3.7	31.5 a	20.3	21.7	7.5	8.4	0.6
Coca	1.4	3.6	12.9	5.4	3.6	20.7	71.4 a	19.9	103.7 a	77.9 a	5.0	2.3	42.2 a	17.1	60.5 a	24.3	0.3
Mining	44.9 a	0.5	38.5 a	12.7	0.5	119.8 a	26.9 a	47.1 a	45.8 a	11.5	11.6	66.3 a	75.2 a	7.2	65.6 a	19.9	3.1
Oil	36.9 a	1.6	39.7 a	8.9	1.6	109.6 a	27.7 a	17.1	56.6 a	44.7 a	5.6	43.4 a	28.0 a	6.7	43.6	10.3	0.5
Armed conflict	11.4	2.6	47.5 a	11.8	2.6	113.4 a	0.6	6.5	49.4 a	3.2	13.2	113.0 a	75.0 a	4.0	22.4	24.1	3.7

a Scores >25% indicate a possible imbalance for that specific variable.

b A full table including indices of covariate imbalance before and after matching for each department is available in Supporting Information .

At the regional level, the matching procedure that resulted in the greatest covariate similarity between cells in‐ and outside PAs was submatching; an average of 95% of the variables in each region were balanced. This procedure reduced all covariate imbalances to acceptable levels for each biotic region, with the exception of the Andes, where covariate imbalance persisted for distance to roads and distance to mining (Table 2 & Supporting Information). Subsetting and exact subsetting failed to remove covariate imbalance for several variables in all the regions, but exact subsetting performed better (fewer variables were imbalanced after the matching). An average of 76% and 33% of the variables were imbalanced, respectively (Table 2).

The matched unprotected areas for the whole network showed a slight difference in average forest loss (2.61% over 15 years) compared with the overall unprotected areas (2.72%). These differences were evident within individual regions as well; the size of the difference varied depending on the matching procedure (Fig. 2). For the Orinoco region, this difference among matching procedures in the estimates of percent forest loss outside PAs implied a change in the direction of the effect of protection. Specifically, matched unprotected areas had more forest loss than PAs when using the subsetting, but less forest loss when using submatching (Fig. 2).

### Effectiveness of PAs in Preventing Deforestation

**Figure 4:**
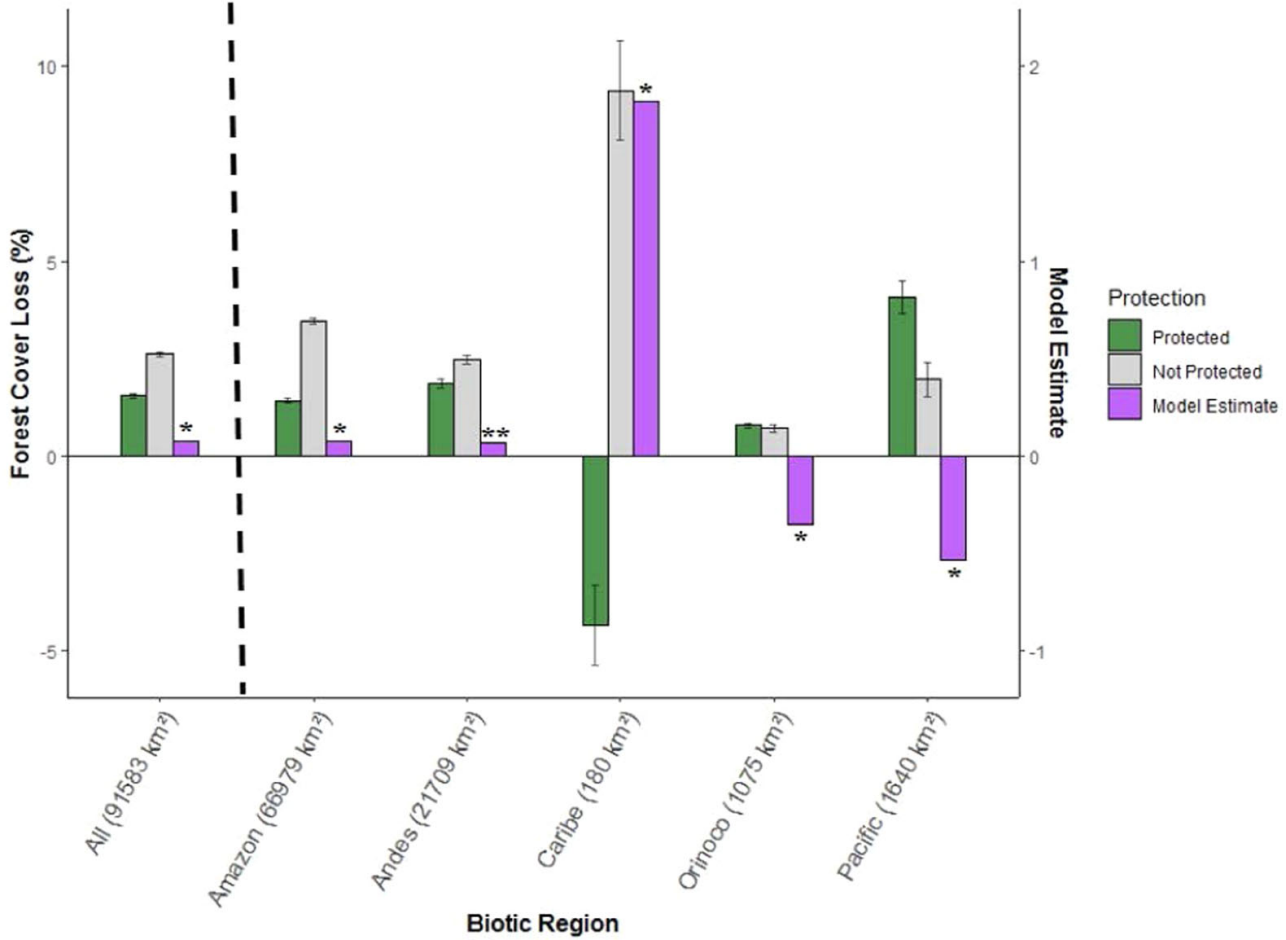
Average percent forest loss in Colombian protected areas (PAs) relative to forest loss in matched unprotected control sites based on the matching protocol that better captured regional variation in the drivers of deforestation and spatial allocation of protected areas (submatching) (numbers in parentheses, square kilometers of PA with forest cover for each biotic region in 2000; green bars, PA; gray bars, matched control sites based on the submatch procedure; negative values, forest gain; black lines, 95% CI; significance of protection effect, ^*^ *p* < 0.05, ^**^ *p* < 0.01). The model‐estimate axis depicts the effect of protection based on the model that better accounted for spatial autocorrelation (model 3).

At the national level, PAs had 1.54% total forest loss compared with 2.61% for unprotected matched areas over 15 years (Fig. 4), a 40% lower forest loss in PAs than in matched unprotected areas. The effect of protection reducing deforestation was significant based on estimates of the best performing model (model 3) (Fig. 4).

At the regional level, protection significantly reduced forest loss in the Amazon, Andes, and Caribe regions (Fig. 4). Protected areas in Caribe were the most effective, but this was also the region with the least forest protected (Figs. 1 & 4). In contrast, protection significantly increased forest loss in the Orinoco and Pacific regions; protection was most ineffective in the Pacific (Fig. 4).

## Discussion

Our results show that residual spatial autocorrelation can overestimate the effectiveness of protection, even when one uses matching analyses. Additionally, the matching procedure used considerably affects the capacity to generate adequate matches when assessing the effectiveness of PAs in regional subsamples.

Ignoring spatial autocorrelation can distort estimates of PA effectiveness in 2 main ways. First, it can wrongly indicate the direction of the effect of protection. In both the Pacific and Orinoco regions, the coefficients of the effect of protection changed from negative to positive when spatial autocorrelation was accounted for. Second, it can inflate the effect of protection, as was the case in the Andes, Caribe, and Amazon regions, where the magnitude of the effect of protection (coefficient value) decreased when spatial autocorrelation was accounted for (Table 1). These results are consistent with findings from ecological studies that compare the performance of spatial and nonspatial models, where spatial autocorrelation had an effect on the magnitude and direction of the coefficient estimates (Dormann 2007; Kühn 2007) and demonstrated the critical importance of accounting for spatial autocorrelation in matching analyses. None of the methods we applied entirely eliminated spatial autocorrelation. Further improvement of the model's performance may be achieved by exploration of alternative procedures that account for variation in spatial autocorrelation patterns, for example, allowing the autocorrelation parameter to vary between control and treatment sites.

Among the three different matching procedures tested to generate matched pairs for each region, submatching generated the best matched controls. Although several studies assessing effectiveness of protection used matching pairs from the overall area of analysis to assess the effectiveness of subsamples (Nelson & Chomitz 2011; Blackman et al. 2015; Bowker et al. 2017), our results showed that these methods can produce poorly matched pairs at the subsample level which in turn reduces the accuracy of the results. This may occur because drivers of deforestation differ among regions, making it difficult to capture these differences in the whole covariate set. We found that generating an independent matching process at each scale of analysis addressed this problem, so we recommend this in future studies.

The PA network in Colombia lost 1.54% of its forest cover from 2000 to 2015. Average forest loss for the matched unprotected areas was 2.61%. This represents 40% less forest loss in PAs. Protected areas in IUCN category VI, which allows sustainable resource extraction, gained forest in the period analyzed. These areas represented 6% of the PA extent in Colombia, but also the majority of protected sites (73%). Many of these small areas were created to protect vital ecosystem services (e.g., water supply), so part of the objectives for these areas involves maintenance of forest and their ecosystem function and active restoration (Colombia Natural National Parks 2019). The fact that these areas are small, which makes them easier to manage, and that frequently they were created to protect specific ecosystem services may explain the patterns found. The PAs with the highest forest lost in the country were instead in category II. This is alarming because this category is supposed to have one of the highest standards of biodiversity protection. Additionally, the areas in this category are mostly large national parks, which makes them difficult to manage (Geldmann et al. 2019).

Protection had an overall statistically significant positive effect on reducing deforestation in the country, but there were important differences among regions in this effect. These differences were likely due to factors such as the number of PAs in each region, the size of PAs, their isolation (which affects the capacity to manage them), and the number of staff in each PA (Geldmann et al. 2019). In several regions where PAs were ineffective in preventing deforestation, investment in improving the effectiveness of the existing PAs should be a priority. However, in regions like Caribe, where PAs were highly effective but deforestation risk outside them was high, investment in creation of more PAs may be a more effective strategy. Finally, in the case of ineffective protection it is important to assess the deforestation rates because ineffective PAs with high deforestation should be considered an investment priority, as is the case of those in the Pacific region.

Protected areas in the Amazon, Andes, and Caribe significantly reduced deforestation. However, PAs in the Amazon reduced deforestation the least and had the highest net forest loss (Fig. 1). This region has the largest PAs in the country, and they are relatively isolated, so their management is particularly difficult. Recent assessments of forest‐cover loss show a dramatic increase in deforestation in the region (Clerici et al. 2018). Some of the agents of deforestation include natural and human caused fires, which have been increasing in Colombia, particularly inside PAs in the Amazon region, where illegal armed groups are present (Armenteras et al. 2019). Elucidating and halting the mechanisms through which FARC demobilization has enabled a new rush of fires in Colombian PAs is needed.

Protection was most effective in reducing deforestation in the Caribe region. The Caribe is one of the regions with the fewest remaining natural areas (Forero‐Medina & Joppa 2010) and the lowest PA coverage (Fig. 1). The effectiveness of the region's PAs, combined with their current low coverage, suggests that creation of new PAs (potentially combined with active restoration of degraded ecosystems) should be a priority in this region.

Protected areas in the Orinoco and Pacific region were ineffective at reducing deforestation, which continued to occur at rates of 0.05% and 0.27% a year within PAs, respectively. The Pacific region is a biodiversity hotspot (Myers et al. 2000), and deforestation is related to mining and illegal crop cultivation (Cagan 2014; Negret et al. 2019). Redressing the effectiveness of PAs in these regions should be a priority, especially for the Pacific, where PA coverage of forest ecosystems remains relatively low (Fig. 1), despite its high biodiversity and its richness of endemic species (Myers et al. 2000). It is therefore particularly important to safeguard the few PAs that exist.

We stress the importance of considering spatial autocorrelation in assessing PA effectiveness because its presence can distort conservation assessments. Also, we highlight the importance of generating independent matching for different spatial dimensions of analysis to account for regional differences in the drivers of deforestation and spatial allocation of PAs. Specific modeling settings can help investigate differences in the effectiveness of PAs across regions and across spatial scales. Robust estimates of PA effectiveness are essential to inform conservation intervention in different contexts so as to elucidate which PAs are effective and which are not in their respective local contexts and to support decisions on whether to better manage existing PAs or create new ones. This method can be applied to any country or region to robustly assess the performance of PAs at preventing ecosystem degradation.

## Supporting information

Variables associated with deforestation and park location in Colombia (Appendix S1), a complete list of indices of covariate imbalance before and after the matching process for each matching method (Appendix S2), and forest‐cover loss from 2000 to 2015 in Colombia's protected areas grouped by IUCN category (Appendix S3) are available online. The authors are solely responsible for the content and functionality of these materials. Queries (other than absence of the material) should be directed to the corresponding author.
