# Supplementary material for: Effects of spatial autocorrelation and sampling design on estimates of protected area effectiveness
Source: Conserv Biol. 2020 Aug 13;34(6):1452–62. doi: 10.1111/cobi.13522 (PMC7885028; doi:10.1111/cobi.13522)
Supplement: Supplementary file 1 — Variables associated with deforestation and park location in Colombia (Appendix S1), a complete list of indices of covariate imbalance before and after the matching process for each matching method (Appendix S2), and forest‐cover loss from 2000 to 2015 in Colombia's protected areas grouped by IUCN category (Appendix S3) are available online. The authors are solely responsible for the content and functionality of these materials. Queries (other than absence of the material) should be directed to the corresponding author. [file COBI-34-1452-s001.docx]

**Supplementary material:**

**SI Table 1.** Variables that have been found to be associated with deforestation and park location in Colombia that were used for the matching analysis.

| Variable type | Variable Description | Source |
| --- | --- | --- |
| Initial forest cover | Percentage of forest cover in 2000 | This study |
| Biotic region | Biotic Regions of Colombia | Humboldt Institute |
| Elevation | DEM Colombia | Humboldt Institute |
| Slope | DEM Colombia | Humboldt Institute |
| Departments | Departments of Colombia | Geographical information system for planning and territorial ordering - SIGOT |
| Population density | Population density by km² | National Administrative Department of Statistics - DANE |
| Distance to Rivers | Distance to nearest navigable river | Institute of Hydrology, Meteorology and Environmental Studies - IDEAM |
| Distance to Roads | Distance to nearest paved road | Geographic Institute Agustín Codazzi - IGAC |
| Distance to Roads | Distance to nearest unpaved road | Geographic Institute Agustín Codazzi - IGAC |
| Coca | Distance to nearest coca plantation | Integrated illicit crops monitoring system -SIMCI |
| Mining | Distance to nearest mining concession | National Mining Agency - ANM |
| Oil exploitation | Distance to nearest exploited oil well | National Hydrocarbon Agency - ANH |
| Armed conflict Intensity | Number of armed actions per area | Negret et al. 2019 |

**SI Table 2.** Indices of covariate imbalance, before and after the matching process for **(a)** an initial matching analysis at a national scale and; A matching analysis at a national scale and then a separate assessment of each regions protected areas and their respective matched controls (sub-setting). A matching analysis at a national scale with exact matching for regions and then a separate assessment of each regions protected areas and their respective matched controls (exact sub-setting). And an individual matching analysis for each region (sub-matching). **(b)** Protected areas in the Amazon region. **(c)** Protected areas in the Andes region. **(d)** Protected areas in the Caribe region. **(e)** Protected areas in the Orinoco region. **(f)** Protected areas in the Pacific region. In bold variables with covariate imbalance.

**a)**

| **All** | Before Matching | After Matching |
| --- | --- | --- |
|  |  |  |
|  |  |  |
| Initial forest Cover | **75.7** | 2.5 |
| Elevation | **26.4** | 5.3 |
| Slope | 17.0 | 5.9 |
| Population density | 2.9 | 0.2 |
| Rivers | 16.4 | 1.3 |
| Paved roads | **25.7** | 6.1 |
| Unpaved roads | 23.9 | 1.0 |
| Coca | 1.4 | 2.9 |
| Mining | **44.9** | 2.4 |
| Oil | **36.9** | 1.1 |
| Armed conflict | 11.4 | 2.8 |
|  |  |  |
| **Departments** |  |  |
| Amazonas | 8.4 | 1.9 |
| Antioquia | 16.3 | 1.4 |
| Arauca | 3.9 | 6.5 |
| Atlantico | 8.1 | Na |
| Bogota | 7.7 | 1.1 |
| Bolivar | 22.8 | 0.2 |
| Boyaca | 2.4 | 1.5 |
| Caldas | 9.1 | 0.3 |
| Caqueta | **45.7** | 8.0 |
| Casanare | **29.7** | 1.4 |
| Cauca | 15.0 | 1.1 |
| Cesar | 16.0 | 0.5 |
| Choco | 15.4 | 2.4 |
| Cordoba | 7.6 | 9.8 |
| Cundinamarca | 5.8 | 0.9 |
| Guainia | 15.4 | 2.1 |
| Guaviare | **28.2** | 9.3 |
| Huila | 4.7 | 0.6 |
| La Guajira | 0.9 | 4.9 |
| Magdalena | 3.0 | 2.9 |
| Meta | 13.2 | 3.6 |
| Nariño | 12.9 | 0.6 |
| Norte de Santander | 0.5 | 7.3 |
| Putumayo | 12.8 | 3.4 |
| Quindio | 5.0 | 0.4 |
| Risaralda | 0.3 | 0.1 |
| Santander | 22.5 | 0.4 |
| Sucre | 13.2 | 0.2 |
| Tolima | 1.0 | 2.0 |
| Valle del Cauca | 11.6 | 1.0 |
| Vaupes | **32.1** | 0.3 |
| Vichada | 16.3 | 11.4 |
|  |  |  |
| **Biotic Regions** |  |  |
| Amazon | **58.1** | 5.1 |
| Orinoco | **46.0** | 0.2 |
| Pacific | 18.0 | 1.9 |
| Andes | 3.4 | 5.0 |
| Caribe | **44.9** | 1.3 |

**b)**

| **Amazon** | Before Matching | After Matching | | |
| --- | --- | --- | --- | --- |
|  |  | *Sub-setting* | *Exact Sub-setting* | *Sub-Matching* |
|  |  |  |  |  |
| Initial forest Cover | **75.7** | **36.6** | 5.6 | 3.7 |
| Elevation | **26.4** | **68.6** | **29.8** | 13.4 |
| Slope | 17.0 | **55.9** | 16.5 | 8.4 |
| Population density | 2.9 | 17.1 | 6.8 | 1.3 |
| Rivers | 16.4 | 3.3 | 18.0 | 3.7 |
| Paved roads | **25.7** | **27.6** | 1.0 | 5.1 |
| Unpaved roads | 23.9 | **38.3** | 3.7 | 0.8 |
| Coca | 1.4 | 12.9 | 5.4 | 3.6 |
| Mining | **44.9** | **38.5** | 12.7 | 0.5 |
| Oil | **36.9** | **39.7** | 8.9 | 1.6 |
| Armed conflict | 11.4 | **47.5** | 11.8 | 2.6 |
|  |  |  |  |  |
| **Departments** |  |  |  |  |
| Amazonas | 8.4 | 19.1 | 3.4 | 4.3 |
| Antioquia | 16.3 | 22.1 | NA | NA |
| Arauca | 3.9 | 9.7 | NA | NA |
| Atlantico | 8.1 | NA | NA | NA |
| Bogota | 7.7 | 12.3 | NA | NA |
| Bolivar | 22.8 | 1.1 | NA | NA |
| Boyaca | 2.4 | 16.8 | NA | NA |
| Caldas | 9.1 | 3.6 | NA | NA |
| Caqueta | **45.7** | **34.5** | 18.4 | 11.3 |
| Casanare | **29.7** | 3.6 | NA | NA |
| Cauca | 15.0 | 8.6 | 0.5 | NA |
| Cesar | 16.0 | 7.3 | NA | NA |
| Choco | 15.4 | 12.6 | NA | NA |
| Cordoba | 7.6 | 17.2 | NA | NA |
| Cundinamarca | 5.8 | 14.6 | NA | NA |
| Guainia | 15.4 | 15.9 | 11.7 | 0.6 |
| Guaviare | **28.2** | 1.6 | 17.0 | 9.3 |
| Huila | 4.7 | 14.2 | NA | NA |
| La Guajira | 0.9 | 13.7 | NA | NA |
| Magdalena | 3.0 | 18.6 | NA | NA |
| Meta | 13.2 | 9.4 | **25.5** | 3.5 |
| Nariño | 12.9 | 9.7 | NA | NA |
| Norte de Santander | 0.5 | **26.5** | NA | NA |
| Putumayo | 12.8 | 3.4 | 2.1 | 5.4 |
| Quindio | 5.0 | 1.3 | NA | NA |
| Risaralda | 0.3 | 8.2 | NA | NA |
| Santander | 22.5 | 2.4 | NA | NA |
| Sucre | 13.2 | 0.9 | NA | NA |
| Tolima | 1.0 | 22.2 | NA | NA |
| Valle del Cauca | 11.6 | **25.5** | NA | NA |
| Vaupes | **32.1** | 4.1 | 0.1 | 0.3 |
| Vichada | 16.3 | 41.5 | **27.1** | NA |

**c)**

| **Andes** | Before Matching | After Matching | | |
| --- | --- | --- | --- | --- |
|  |  | *Sub-setting* | *Exact Sub-setting* | *Sub-Matching* |
|  |  |  |  |  |
| Initial forest Cover | **75.7** | **50.0** | 12.7 | 1.6 |
| Elevation | **26.4** | **124.6** | 4.4 | 3.1 |
| Slope | 17.0 | **112.5** | 2.9 | 5.9 |
| Population density | 2.9 | 21.2 | 7.3 | 3.5 |
| Rivers | 16.4 | 12.5 | **60.5** | 0.2 |
| Paved roads | **25.7** | **115.3** | **41.5** | **29.7** |
| Unpaved roads | 23.9 | **94.6** | 0.7 | **29.4** |
| Coca | 1.4 | 20.7 | **71.4** | 19.9 |
| Mining | **44.9** | **119.8** | **26.9** | **47.1** |
| Oil | **36.9** | **109.6** | **27.7** | 17.1 |
| Armed conflict | 11.4 | **113.4** | 0.6 | 6.5 |
|  |  |  |  |  |
| **Departments** |  |  |  |  |
| Amazonas | 8.4 | **38.3** | NA | NA |
| Antioquia | 16.3 | **25.1** | 1.9 | 7.6 |
| Arauca | 3.9 | **27.6** | 24.6 | 20.7 |
| Atlantico | 8.1 | NA | NA | NA |
| Bogota | 7.7 | 12.9 | 3.1 | 0.8 |
| Bolivar | 22.8 | 1.6 | 0.8 | NA |
| Boyaca | 2.4 | 23.6 | 1.1 | 4.3 |
| Caldas | 9.1 | 6.9 | 1.4 | 3.2 |
| Caqueta | **45.7** | 62.1 | 21.7 | 1.4 |
| Casanare | **29.7** | 0.9 | 0.4 | 0.4 |
| Cauca | 15.0 | 15.9 | 3.2 | 11.7 |
| Cesar | 16.0 | 11.2 | 5.1 | 3.2 |
| Choco | 15.4 | 5.7 | 13.0 | 2.7 |
| Cordoba | 7.6 | **39.6** | **31.6** | **25.9** |
| Cundinamarca | 5.8 | 21.3 | 1.2 | 8.4 |
| Guainia | 15.4 | **53.6** | NA | NA |
| Guaviare | **28.2** | **51.6** | NA | NA |
| Huila | 4.7 | 19.7 | 0.5 | 8.2 |
| La Guajira | 0.9 | 22.4 | 19.3 | 13.8 |
| Magdalena | 3.0 | 21.0 | 7.8 | 12.2 |
| Meta | 13.2 | 2.7 | 6.8 | 3.1 |
| Nariño | 12.9 | 7.4 | 2.8 | 9.5 |
| Norte de Santander | 0.5 | 19.8 | 11.4 | 15.1 |
| Putumayo | 12.8 | 22.6 | **28.0** | 9.2 |
| Quindio | 5.0 | 2.9 | 0.5 | 0.4 |
| Risaralda | 0.3 | 10.7 | 0.5 | 5.5 |
| Santander | 22.5 | 6.4 | 4.1 | 0.9 |
| Sucre | 13.2 | 3.7 | 1.4 | NA |
| Tolima | 1.0 | **25.0** | 4.6 | 3.8 |
| Valle del Cauca | 11.6 | 21.9 | 1.0 | 5.6 |
| Vaupes | **32.1** | 4.3 | NA | NA |
| Vichada | 16.3 | **33.5** | NA | NA |

**d)**

|  | Before Matching | After Matching | | |
| --- | --- | --- | --- | --- |
| **Caribe** |  | *Sub-setting* | *Exact Sub-setting* | *Sub-Matching* |
|  |  |  |  |  |
| Initial forest Cover | **75.7** | **29.9** | **30.6** | 16.2 |
| Elevation | **26.4** | **83.5** | 1.0 | 2.9 |
| Slope | 17.0 | **26.4** | 9.2 | 6.8 |
| Population density | 2.9 | 4.6 | 22.3 | 9.1 |
| Rivers | 16.4 | 14.2 | **46.3** | 12.8 |
| Paved roads | **25.7** | **68.0** | **85.7** | 12.1 |
| Unpaved roads | 23.9 | **51.1** | 22.9 | 3.7 |
| Coca | 1.4 | **103.7** | **77.9** | 5.0 |
| Mining | **44.9** | **45.8** | 11.5 | 11.6 |
| Oil | **36.9** | **56.6** | **44.7** | 5.6 |
| Armed conflict | 11.4 | **49.4** | 3.2 | 13.2 |
|  |  |  |  |  |
| **Departments** |  |  |  |  |
| Amazonas | 8.4 | **49.5** | NA | NA |
| Antioquia | 16.3 | **38.3** | 21.4 | NA |
| Arauca | 3.9 | 20.9 | NA | NA |
| Atlantico | 8.1 | NA | NA | NA |
| Bogota | 7.7 | NA | NA | NA |
| Bolivar | 22.8 | 9.9 | 8.6 | 0.9 |
| Boyaca | 2.4 | 23.8 | NA | NA |
| Caldas | 9.1 | 15.7 | NA | NA |
| Caqueta | **45.7** | **34.2** | NA | NA |
| Casanare | **29.7** | 12.8 | NA | NA |
| Cauca | 15.0 | **28.0** | NA | NA |
| Cesar | 16.0 | 0.0 | 6.8 | 2.6 |
| Choco | 15.4 | **34.8** | NA | NA |
| Cordoba | 7.6 | 12.8 | **109.0** | NA |
| Cundinamarca | 5.8 | 23.8 | NA | NA |
| Guainia | 15.4 | **28.0** | NA | NA |
| Guaviare | **28.2** | 14.4 | NA | NA |
| Huila | 4.7 | 21.4 | NA | NA |
| La Guajira | 0.9 | **76.9** | 8.9 | 4.6 |
| Magdalena | 3.0 | **136.8** | **66.9** | 4.9 |
| Meta | 13.2 | **34.5** | NA | NA |
| Nariño | 12.9 | **32.1** | NA | NA |
| Norte de Santander | 0.5 | 24.3 | 7.8 | NA |
| Putumayo | 12.8 | 15.1 | NA | NA |
| Quindio | 5.0 | NA | NA | NA |
| Risaralda | 0.3 | 10.1 | NA | NA |
| Santander | 22.5 | 8.9 | 0.0 | 2.4 |
| Sucre | 13.2 | **30.2** | 19.6 | 15.4 |
| Tolima | 1.0 | **26.8** | 4.5 | NA |
| Valle del Cauca | 11.6 | 18.2 | NA | NA |
| Vaupes | **32.1** | 17.6 | NA | NA |
| Vichada | 16.3 | **57.5** | NA | NA |

**e)**

| **Orinoco** | Before Matching | After Matching | | |
| --- | --- | --- | --- | --- |
|  |  | *Sub-setting* | *Exact Sub-setting* | *Sub-Matching* |
|  |  |  |  |  |
| Initial forest Cover | **75.7** | **61.7** | 5.4 | 3.2 |
| Elevation | **26.4** | **66.7** | 17.0 | 0.5 |
| Slope | 17.0 | **68.7** | 9.9 | 0.1 |
| Population density | 2.9 | **27.4** | **36.5** | 2.4 |
| Rivers | 16.4 | **54.4** | **143.8** | 5.0 |
| Paved roads | **25.7** | **225.2** | **65.4** | 0.7 |
| Unpaved roads | 23.9 | **31.5** | 20.3 | 21.7 |
| Coca | 1.4 | 2.3 | **42.2** | 17.1 |
| Mining | **44.9** | **66.3** | **75.2** | 7.2 |
| Oil | **36.9** | **43.4** | **28.0** | 6.7 |
| Armed conflict | 11.4 | **113.0** | **75.0** | 4.0 |
|  |  |  |  |  |
| **Departments** |  |  |  |  |
| Amazonas | 8.4 | **47.7** | NA | NA |
| Antioquia | 16.3 | **40.6** | NA | NA |
| Arauca | 3.9 | **25.2** | **65.6** | 2.8 |
| Atlantico | 8.1 | NA | NA | NA |
| Bogota | 7.7 | NA | NA | NA |
| Bolivar | 22.8 | 1.9 | NA | NA |
| Boyaca | 2.4 | 19.8 | 19.7 | NA |
| Caldas | 9.1 | 9.7 | NA | NA |
| Caqueta | **45.7** | **40.9** | NA | NA |
| Casanare | **29.7** | 4.4 | 0.3 | 5.4 |
| Cauca | 15.0 | **29.0** | NA | NA |
| Cesar | 16.0 | 16.2 | NA | NA |
| Choco | 15.4 | **37.4** | NA | NA |
| Cordoba | 7.6 | **30.8** | NA | NA |
| Cundinamarca | 5.8 | 21.6 | 15.3 | 0.0 |
| Guainia | 15.4 | 23.4 | 17.6 | NA |
| Guaviare | **28.2** | 12.8 | 7.7 | NA |
| Huila | 4.7 | 20.1 | NA | NA |
| La Guajira | 0.9 | 19.5 | NA | NA |
| Magdalena | 3.0 | 22.9 | NA | NA |
| Meta | 13.2 | 23.3 | **41.7** | 0.8 |
| Nariño | 12.9 | **33.2** | NA | NA |
| Norte de Santander | 0.5 | 22.1 | NA | NA |
| Putumayo | 12.8 | 12.7 | NA | NA |
| Quindio | 5.0 | 4.9 | NA | NA |
| Risaralda | 0.3 | 8.5 | NA | NA |
| Santander | 22.5 | 16.0 | NA | NA |
| Sucre | 13.2 | 8.5 | NA | NA |
| Tolima | 1.0 | 23.7 | NA | NA |
| Valle del Cauca | 11.6 | 7.5 | NA | NA |
| Vaupes | **32.1** | 9.9 | NA | NA |
| Vichada | 16.3 | **267.2** | **94.4** | NA |

**f)**

| **Pacific** | Before Matching | After Matching | | |
| --- | --- | --- | --- | --- |
|  |  | *Sub-setting* | *Exact Sub-setting* | *Sub-Matching* |
|  |  |  |  |  |
| Initial forest Cover | **75.7** | **53.3** | 15.2 | 3.2 |
| Elevation | **26.4** | **57.1** | 0.5 | 5.4 |
| Slope | 17.0 | 24.1 | 2.1 | 5.5 |
| Population density | 2.9 | 11.1 | 6.2 | 4.7 |
| Rivers | 16.4 | **81.3** | 3.0 | 1.5 |
| Paved roads | **25.7** | **78.9** | 3.1 | 2.2 |
| Unpaved roads | 23.9 | 7.5 | 8.4 | 0.6 |
| Coca | 1.4 | **60.5** | 24.3 | 0.3 |
| Mining | **44.9** | **65.6** | 19.9 | 3.1 |
| Oil | **36.9** | **43.6** | 10.3 | 0.5 |
| Armed conflict | 11.4 | 22.4 | 24.1 | 3.7 |
|  |  |  |  |  |
| **Departments** |  |  |  |  |
| Amazonas | 8.4 | **52.7** | NA | NA |
| Antioquia | 16.3 | 16.1 | 2.8 | 5.3 |
| Arauca | 3.9 | 22.2 | NA | NA |
| Atlantico | 8.1 | NA | NA | NA |
| Bogota | 7.7 | 5.4 | NA | NA |
| Bolivar | 22.8 | 4.2 | NA | NA |
| Boyaca | 2.4 | **25.4** | NA | NA |
| Caldas | 9.1 | 9.9 | NA | NA |
| Caqueta | **45.7** | **36.2** | NA | NA |
| Casanare | **29.7** | NA | NA | NA |
| Cauca | 15.0 | 11.4 | 10.2 | 0.8 |
| Cesar | 16.0 | 12.3 | NA | NA |
| Choco | 15.4 | **110.9** | 1.4 | 3.1 |
| Cordoba | 7.6 | 20.9 | NA | NA |
| Cundinamarca | 5.8 | 23.5 | NA | NA |
| Guainia | 15.4 | **42.5** | NA | NA |
| Guaviare | **28.2** | **32.4** | NA | NA |
| Huila | 4.7 | 17.8 | NA | NA |
| La Guajira | 0.9 | 23.3 | NA | NA |
| Magdalena | 3.0 | 22.5 | NA | NA |
| Meta | 13.2 | **44.1** | NA | NA |
| Nariño | 12.9 | **44.7** | 0.8 | 2.7 |
| Norte de Santander | 0.5 | 23.8 | NA | NA |
| Putumayo | 12.8 | 22.3 | NA | NA |
| Quindio | 5.0 | 3.4 | NA | NA |
| Risaralda | 0.3 | 9.6 | NA | NA |
| Santander | 22.5 | 16.2 | NA | NA |
| Sucre | 13.2 | 7.2 | NA | NA |
| Tolima | 1.0 | 20.2 | NA | NA |
| Valle del Cauca | 11.6 | **61.1** | 1.6 | 2.2 |
| Vaupes | **32.1** | 12.1 | NA | NA |
| Vichada | 16.3 | **48.0** | NA | NA |

**
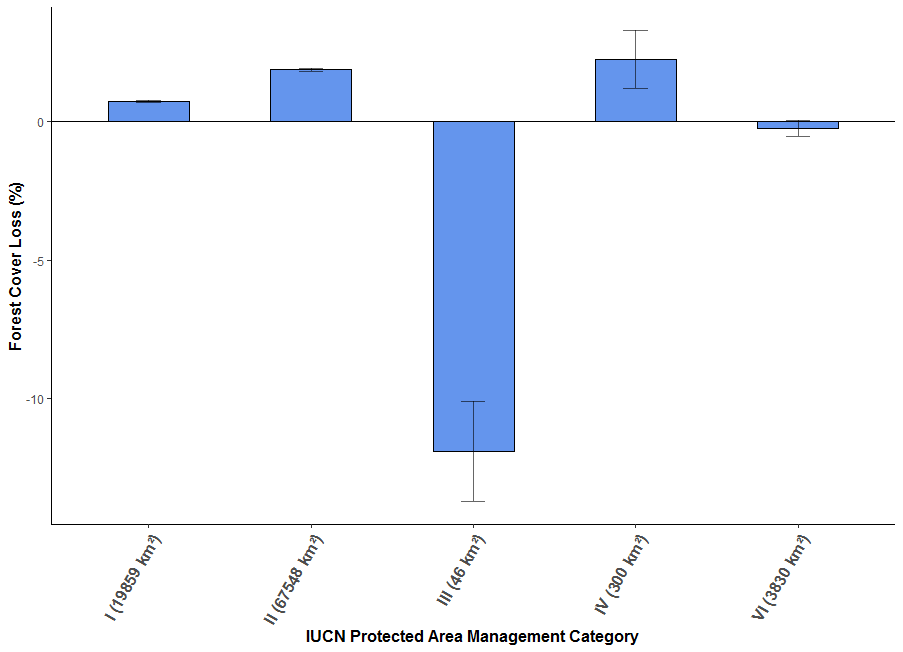
**

**SI Figure 1.** Forest cover loss from 2000 to 2015 in Colombia’s protected areas grouped by IUCN category. Positive values represent forest loss and negative values forest gain. Black lines depict 95% confidence intervals. Numbers in parenthesis are the area with forest cover under each IUCN category in 2000.
